# On the Shoulders of Giants: The Specialist Surgeon Workforce in East, Central and Southern Africa

**DOI:** 10.1002/wjs.12544

**Published:** 2025-03-06

**Authors:** Dominique Vervoort

**Affiliations:** ^1^ Division of Cardiac Surgery University of Toronto Toronto Canada; ^2^ Institute of Health Policy, Management and Evaluation University of Toronto Toronto Canada

**Keywords:** COSECSA, global health, global surgery, surgical workforce

Surgical care is an indispensable component of robust and resilient health systems, closely linked with health system responsiveness and overall population health. The *Lancet Commission on Global Surgery* proposed surgical workforce targets of 20–40 surgeons, anesthesiologists, and obstetricians per 100,000 population to meet the emergency and essential surgical needs of countries' population [1]. Although not explicitly describing the relative needs of surgeon specialists or subspecialists, these targets reflect the need for considerable strengthening of the global surgical workforce to a minimum of approximately 10–20 surgeons per 100,000 population, with subspecialty numbers varying depending on local disease burdens.

In 2016, the first comprehensive report of the surgeon specialist workforce in the region of the College of Surgeons of East, Central and Southern Africa (COSECSA) was published, now updated by Osei‐Kuffour et al. to reflect more contemporary realities in 12 of the 14 COSECSA countries [2]. During the past decade, there has been an increase in the surgeon specialist workforce in the COSECSA region by 42% (from 1690 in 2015 to 2555 surgeons in 2022). Relative to the rapid population growth in the region, this represents an increase from 0.53 to 0.59 surgeons per 100,000 population. Great variation exists in surgeon density among countries, with an 18‐factor difference between the highest (3.97 surgeons per 100,000 population in Namibia) and lowest workforce densities (0.22 surgeons per 100,000 population in Mozambique). Meanwhile, the men‐to‐women ratio among surgeons remained constant at 9:1 (9.2% in 2015 vs. 9.6% in 2022). Most (78%) surgeons work in the country of their primary qualification, whereas 42% of surgeons work in less densely populated regions.

The COSECSA developments in the surgeon workforce must be seen as a tremendous success, despite overall numbers still dictating challenges ahead in meeting the surgical needs of the region's population. The training model developed by COSECSA is unique and highly successful, reflected by many positive experiences of surgeons and trainers alike as well as the low rates of brain drain [3]. The additional provision of academic opportunities, continued medical education, and a professional network further support local surgeons in ways often not seen in other regions [4]. This helps explain why COSECSA was able to train, welcome, and maintain nearly 1000 additional surgeons in a little over 5 years. Moreover, the considerable number of surgeons working in district hospitals and more remote areas suggest the effectiveness of current paradigms in expanding emergency and essential surgical care services outside large population centers.

Various challenges do remain, each presenting its unique opportunities. First, population growth is occurring at a tremendous pace, resulting in the absolute number gains in surgeons to be mitigated by population‐level increases. On the one hand, this indicates an immediate need for surgeon specialists in areas affecting younger (e.g., pediatric surgery for congenital anomalies), more mobile (e.g., trauma surgery for injuries), reproductive (e.g., obstetric surgery for pregnancies), marginalized (e.g., cardiac surgery for congenital and rheumatic heart disease), and surging conditions (e.g., surgical oncology for malignancies). On the other hand, this reality calls for attempts to sustainably and feasibly scale current training models, requiring sufficient political buy‐in to ensure the necessary funding and development of further and more decentralized teaching hospitals. Second, subspecialty numbers do not inherently reflect population needs based on existing disease burdens (e.g., availability of cardiothoracic surgeons vs. cardiovascular and thoracic disease burdens), whereas women's representation is low and particularly limited in subspecialties. For example, for cardiothoracic surgery, the authors report 69 surgeons, of which only two (2.9%) are women. Comparatively, across sub‐Saharan Africa, 4.9% are women [5]. Support of locoregional efforts, such as Women in Surgery Africa, is critical for both current and future women surgeons. As the COSECSA workforce grows, support should be tailored to meet the challenges and needs of women surgeons in different subspecialties, similar to efforts by global subspecialty professional societies. Third, an operation cannot happen in isolation, as safe anesthesia is a key to ensuring safe surgery, whereas nursing availability is a common barrier toward ensuring optimal care delivery. In the case of certain subspecialties, further workforce requirements are necessary and indispensable (e.g., intensive care personnel, technicians, and perfusionists). This will require close communication and interdisciplinary collaboration with other such societies. Similarly, resource availability and surgical supply chains must be strengthened to allow surgeons to safely increase surgical volumes within the region, inviting support by (preferably regional) industry stakeholders. Lastly, generating accurate and up‐to‐date workforce numbers is critical to inform future locoregional health policies and the impact of training programs and existing national health policies. In the era of artificial intelligence, particularly with large language models, opportunities exist to reduce data and administrative burdens for the College and countries alike. Complementing these opportunities are the growth in national surgical, obstetric, and anesthesia plans across most COSECSA countries, the momentum by global surgical stakeholders, such as the Global Surgery Foundation and Royal College of Surgeons in Ireland, and early trainee engagement through groups such as InciSioN (the International Student Surgical Network), the University of Global Health Equity, and more. Together, these provide hope that future workforce reports will show even greater progress.

Each current and future surgeon in the region stands on the shoulders of COSECSA giants leading the way in strengthening the surgeon workforce for almost half a billion people. Efforts to expand and support the surgical workforce in the 14 COSECSA countries are well underway and should be celebrated while exploring avenues to effectively, sustainably, and feasibly scale existing mechanisms to meet rapid population growth into the future.

## Author Contributions

**Dominique Vervoort:** conceptualization, investigation, writing – original draft, writing – review and editing.

## Disclosure

The author has nothing to report.

## Conflicts of Interest

The author declares no conflicts of interest.
